# On Pneumothorax

**Published:** 1895-09

**Authors:** Charles Lewis Allen

**Affiliations:** New York, Instructor in Medicine New York Polyclinic; Attending Physician Good Samaritan Dispensary; Assistant Physician Lebanon Hospital


### ON PNEUMOTHORAX.

By CHARLES LEWIS ALLEN, M.D., New York,

Instructor in Medicine New York Polyclinic; Attending Physician Good Samaritan Dispensary; Assistant Physician Lebanon Hospital.

By pneumothorax is meant the presence of gas in the pleural cavity. Gas is, however, seldom present alone, but is usually accompanied by liquid, either serous, purulent, or, very rarely, bloody, giving rise respectively to hydropneumothorax, pyopneumothorax, and hemopneumothorax. These conditions are not very uncommon, but are, nevertheless, sufficiently infrequent, and sometimes obscure enough to render diagnosis difficult, unless one is alive to the possibility of their occurrence, and is familiar with their physical signs. May these considerations, and the fact that I have had recently the opportunity of observing three very difficult and yet very characteristic cases, be my excuse for presenting, on this subject, a short paper.

Although it has been pretty well proven that gas can be generated in the pleural cavity by the decomposition of an exudate, in the vast majority of cases its presence is due to the establishment of communication between this cavity and the external air through the chest wall, through the lung, or through some portion of the digestive tract. Cases produced by external injury belong to the domain of surgery, and do not come within the scope of this paper. Should the wall of the esophagus become adherent to the pleura and ulceration and perforation occur, a pneumothorax may be set up. Such an occurrence must of course be rare, but its possibility was brought forcibly before me by the following case, from the practice of a friend, by whose courtesy I was permitted to attend the autopsy:

A man about twenty-six years old, whose history, beyond the fact that he had been ailing for some time, we were unable to learn, began suddenly to vomit blood and died before a physician could reach him. The autopsy showed that the esophagus had become adherent to the pericardium, the two layers of which were fused together at this point, and ulceration and perforation into the right auricle of the heart had occurred. The liver and spleen showed some grave changes suggesting syphilis, but through an unfortunate accident the specimens were lost, and no microscopical examination was made; hence the cause of the ulceration was not determined. In the case of communication between the pleural cavity and the stomach or intestine, adherence to and perforation of the diaphragm must of course occur. The great majority of cases, however, arise from perforation from the lung. This may occur in several ways. An air vesicle may be ruptured by a sudden strain, such as a violent fit of coughing (as in pertussis) or a sudden and severe muscular effort. This is more likely to happen in the case of a lung already emphysematous. An empyema sometimes breaks through the lung into the branches, setting up a pleuro-bronchial fistula and a pyopneumothorax. By far the most common method of production, however, is the breaking down and perforation of a focus of disease, situated just beneath the pleura in the lung. This may be an area of pulmonary apoplexy, a lung abscess or gangrenous sprout, a cancerous nodule, an echinococcus, or a dilated bronchus. The immense majority of cases, however, are due to the softening and giving way of a caseous nodule, or to the rupture of the wall of a tuberculous cavity, ninety per cent. of all cases being stated to be caused in this manner. Once formed, the opening may remain patulous, may become sealed up, or a false membrane formed over it may act as a valve, giving rise to three varieties—open, closed, and valvular pneumothorax.

The gas may occupy more or less of the pleural cavity, depending upon the size of the opening, the amount of liquid present, etc. Where old pleural adhesions are present the gas may be encysted, occupying only a limited space as shown in one of the cases whose history is given below. As already stated, it is very rare to find a pure pneumothorax, the gas being almost always accompanied by fluid, serous or purulent, or, in very rare cases, bloody. It has been shown, however, that in case of the admission of air by the rupture of a comparatively healthy lung vesicle, the gas may be absorbed without the production of inflammation and exudation. The gas present is remarkably poor in oxygen and rich in carbon dioxide and nitrogen, an average example being nitrogen eighty-five per cent., carbon dioxide twelve per cent., oxygen three per cent. Where the orifice remains open, however, the carbon dioxide may fall to three or four per cent., and the oxygen may correspondingly increase where decomposing pus is present; sulphuretted hydrogen may occur, and is recognized at once by its odor.

Pneumothorax may begin gradually or suddenly, depending much upon its cause. After severe exertion the patient may be conscious of something giving way, and experience a sensation of pain and tearing in the chest, followed by great dyspnea. The temperature may be somewhat raised, but depends mainly upon the antecedent disease. Upon inspection the affected side may be noticed to move less than the sound one, and may appear bulged, the interspaces of the ribs being filled out. On palpation a total loss of vocal fremitus may be noted. On percussion the note is usually tympanitic, its pitch depending upon the tension of the gas, and approaching flatness where this is very high. There may be noticed also a feeling of great elasticity, as if a rubber air-cushion were beneath the finger. The percussion note transmitted through the chest has usually a peculiar metallic character, which is best brought out by auscultatory percussion in the following way: An assistant (or a bystander) is requested to press a coin (a silver half-dollar is very suitable) firmly upon the front of the chest, at about the second or third intercostal space of the affected side, and to tap on it with another coin. The observer auscultiug behind, will have the sound transmitted to him with a characteristic metallic ring. This sign, which is almost absolutely pathognomonic of pneumothorax, was first described by Trousseau, who called it the "bruit d'airain" (or brassy sound). In this country it is generally spoken of as the "coin test." It does not seem possible to get it, in case of a large lung cavity, and it may be absent in case of an encysted pneumothorax. Where fluid is present, there is dullness over the lower part of the chest, reaching to the upper level of the liquid, and changing with the position of the patient.

On auscultation, the respiratory murmur may be absent over the affected side, or may be heard very faintly and distantly. It has usually a marked amphoric character. Metallic tinkling may be present also, but is variable, appearing and disappearing. This sign, which is also found over large lung cavities, resembles the sound produced by the dropping of pins into a metal dish. The method of its origin has been disputed. It has been attributed to the dripping of mucus from the roof of a cavity, and to the bubbling of air through liquid contained in a cavity, but it seems to the writer—certainly in the majority of cases—to be due to a modification of sound which râles produced in neighboring bronchi undergo in passing across a large cavity filled with air. This is indicated by the fact that not only are râles given a metallic character by the presence of a large air cavity, but the heart sounds may be rendered clear and bell-like, as shown in case No. 2, related below. The matter seems proven beyond a doubt, however, by the experiment of Behier, who filled a rubber balloon with air, closed it, immersed it in water, and had an assistant blow air through a tube beneath the surface of the water near the balloon. On auscultiug the balloon, Behier heard the sound of the bubbling, having the characteristic metallic quality imparted to it. A sign which is absolutely diagnostic of the presence of gas and liquid in the pleural cavity, is the Hippocratic succussion sound. This is a peculiar splashing, like that heard on shaking a cask containing a small amount of liquid, and is elicited by having some one grasp the patient by the shoulders and give him a sudden shake, while the observer auscults on the affected side. When the liquid is very thick and viscous, this sign may fail, however. The classical signs are tympanitic percussion note, absent or faint respiratory murmur with metallic phenomena, the "bruit d'airain," and the succussion sound. When these are present together the diagnosis may be regarded as certain. The prognosis is always grave, save in those rare cases where the condition is due to the rupture of air vesicles and is not accompanied by inflammation and exudation. The fatal result, however, is usually due to the underlying disease, and may be postponed for some time, even for one or two years in reputed cases. The treatment is purely palliative. The pain and dyspnea may be combated by hypodermic injections of morphine, with spts. etheris co. and other stimulants by the mouth. Inhalations of oxygen may be of some temporary benefit. Should the volume of the gas be very great and the pressure in the chest very high, a portion of the gas may be evacuated by a very fine trocar. If there is much liquid present, aspiration may be resorted to, but it must be remembered that sudden lowering of the pressure in the pleural cavity is much more dangerous here than in a simple pleurisy, for if the perforation in the lung has become closed, it may be forced open again, adhesions may be broken down, and hemorrhage may result. Professor Potain, of Paris, has suggested the injection of sterilized air as the liquid is withdrawn, and has devised an apparatus for this purpose which he states has been used with good results. Pyopneumothorax will require the surgical measures which are appropriate in simple empyema.

To this short clinical sketch I add the histories of three cases, with comments upon them. The first two I saw through the courtesy of my friend, Dr. John Kephé, who has kindly permitted me to utilize them; but as they soon passed out of his hands the histories are necessarily imperfect. The third case I was able to follow up until his discharge from the hospital.

1. ANN S.—Nineteen; a large, well-built woman; family history unobtainable; had a cough for a year, but was able to work. On March 8, 1893, she was taken sick with fever, cough, and dyspnea. On examination, there was found, breathing rapid, no change in contour of chest. On right side loss of vocal fremitus, and percussion gave tympanitic note above sixth rib; below this flatness, the level changing with change of position of the patient. Over tympanitic area coin test positive. Respiratory murmur on left side clear; on right side, distant and of amphoric character. No metallic tinkling. Succussion splash easily obtainable, and needle introduced over area of flatness drew clear serous fluid. Patient was kept in bed and treated with small doses of morphine, but dyspnea grew worse and amount of liquid increased. On March 15th about a pint of liquid was withdrawn by aspirator, with some temporary improvement. Her condition then getting gradually worse, she was removed to Mt. Sinai Hospital, where she died about two months later. No autopsy.

2. HENRY L.—Twenty-two; family history strongly tuberculous, one sister died of phthisis. Patient had had a cough and been thin and delicate since pneumonia two years ago. On March 20, 1893, he was taken while at work with severe pain in the chest, followed by dyspnea and free expectoration of very fetid pus. When seen on March 23d, he was suffering from severe dyspnea. Left side of chest markedly bulged out, impulse of heart visible under right nipple. On the left side, absence of vocal fremitus, and percussion gave tympanitic note above the sixth rib; below this complete flatness, the level being altered on change of position. On right side an area of dullness reached from sternum to nipple at cardiac level. Coin test positive on left side. Auscultation showed diffused râles over both lungs. On the left side respiratory murmur weak and distant, and metallic tinkling present. The heart sounds as heard on the left side were peculiarly modified, their character being best illustrated by the sound produced by hammering a piece of iron on an anvil, when heard at some distance. This sound disappeared from time to time, and then returned on change of position of the patient. Succussion splash was obtained. Patient's condition grew worse; a friction murmur synchronous with the heart sound developed over the cardiac area, dyspnea became greater, he had irregular fever and sweating, and developed an anal fistula which discharged very foul pus. He declined any operation, and was sent to St. Joseph's Hospital, where he died soon after. The result of the autopsy could not be learned. It is doubtful if one was obtained.

3. MOSES N.—Nineteen years old; fairly stout, well-built man, one brother died of phthisis. About February, 1894, was sick with fever, cough, and pain in the left side, but got better without medical attendance. On May 21, 1894, after climbing four flights of stairs, he felt a sudden sharp pain in his chest, followed by cough and dyspnea. When seen some hours later, he was short of breath but did not appear very sick. The left side of his chest moved slightly less than the right, and the heart's impulse was not visible. Vocal fremitus was diminished over the whole left side, and was completely lost below the third rib between the sternum and the nipple. Percussion elicited a clear tympanitic note and feeling of elasticity over an area extending from the third to the sixth rib and from nipple to sternum on the left side, the note over the rest of the left lung being vesiculo-tympanitic. Percussing across from left to right, dullness began at the left border of the sternum and extended to the right nipple. This dullness extended vertically on the right side of the sternum, from the third to the sixth rib, the percussion note being normal over the rest of the right lung. Diffuse mucous râles were heard over both lungs, and the respiratory murmur was faint over the tympanitic area on the left side, with no metallic tinkle. To the left of the sternum the heart sounds were weaker than on the right, and the first sound was followed by a peculiar clicking or tearing, somewhat metallic sound, heard most plainly on the left of the sternum, but perceptible in the axilla and at the back near the scapula, but not on the right of the sternum. Coin test negative, temperature normal. On May 22d, the patient being worse, he was sent to the Lebanon Hospital, where he was kept quiet and treated systematically by mild opiates and occasionally counter-irritation. By May 31st vocal fremitus was normal on the left side and the heart sounds were clearly heard, a sharp systolic souffle and a few friction sounds synchronous with respiration being also present on that side. His temperature had not gone above 99.3 F., nor his pulse above 80. On June 3d his physical signs were normal, and he was discharged. I have not been able to see him since, but am told that he is working, but is not very strong and has a cough.

*Comments.*—Case 1 was a hydropneumothorax, and probably resulted from the rupture of a caseous nodule, as the patient had probably had phthisis for a year, and died of it. Aspiration here was productive of no bad results, but of distinct, though temporary, benefit.

Case 2, occurring in a tubercular subject, a pyopneumothorax was most likely produced by the rupture into a bronchus of an old empyema, as evidenced by its sudden production being followed by copious expectoration of foul pus. Some temporary relief might have been given by opening the chest and draining, but this proposition was declined by the patient.

Case 3, the most interesting of all, seems to present the following etiology : The earlier illness was probably a pleurisy, which set up adhesions in such a way as to produce a sac in the part of the pleura near the heart. A rupture of some focus of weakness—most likely a caseous nodule—situated just beneath this sac, caused the effusion into it of air, producing an encysted pneumothorax, which pushed the heart over to the right and compressed a portion of the right lung, giving rise to the physical signs noted. The most remarkable feature of the case seems to be the fact that the air disappeared in so short a time, and did not cause the effusion of liquid. I can see no other diagnosis which would explain the symptoms in the case.

